# Supplementary material for: Learning curve and surgical outcome of robotic assisted colorectal surgery with ERAS program
Source: Sci Rep. 2022 Nov 29;12:20566. doi: 10.1038/s41598-022-24665-w (PMC9709162; doi:10.1038/s41598-022-24665-w)
Supplement: Supplementary file 1 — Supplementary Information 1. [file 41598_2022_24665_MOESM1_ESM.zip › LSC Raw data-Ñ[▒K/112/H_ñw╝╨░O▒Kñσ.pdf]

正式摘要(電子簽章：000000207535041)

W52 / W52 ☐ 英文標題(注意:無英文姓名-請補病人基本檔!) ☐【讀取】憑證

臺中榮民總醫院

出院病歷摘要

轉入號 : 轉出號 :

[redacted]

**(8)**流水編號	
**(9)**入院日期	110/01/06 (2021) 直腸外科 W52 - 155
**(10)**轉科日期	
**(11)**出院日期	110/01/10 (2021) 住院天數計 4 日

診斷

- 入院:1. Rectal cancer
  2.Type 2 DM
  3.Hypopharyngeal cancer, right pyriform sinus, cT2N0M0 s/p OP on Mar. 2019 + CCRT
  4.Thyroid cancer, s/p thyrodectomy on Nov. 2019
- 出院:1. Rectal cancer s/p laparoscopic anterior resection on 2021/1/7
  2.Type 2 DM
  3.Hypopharyngeal cancer, right pyriform sinus, cT2N0M0 s/p OP on Mar. 2019 + CCRT
  4.Thyroid cancer, s/p thyrodectomy on Nov. 2019

主訴

- bloody stool for about 1 month

病史

- This 60 years old male patient has past history of type 2 diabete mellitus and hypopharyngeal cancer, right pyriform sinus, cT2N0M0 s/p OP on Mar. 2019 + CCRT at 彰基 and thyroid cancer, s/p thyrodectomy at 彰基 on Nov. 2019. He noticed that he had bloody stool on last December. There was no other bowel habbit changed or body weight loss. He came to 澄清 for help initially and rectal tumor was found in colonoscopy and malignancy was proved. He came to our hospital for help. Abdominal CT was arranged for staging on 12/30 that no obvious lymphadenopathy or distal metastasis was found. Colonoscopy was arranged on 1/5 for localization. Thus, he was admitted for surgical treatment.

體檢發現

-
  - General appearance :
    - easy looking , conscious clear , without cardiopulmonary distress
    - Vital signs自動帶入date 體溫(BT) 脈搏(P) 呼吸(R) 血壓(BP) 身高(BH) 體重(BW) BMI
      20210106 35.5℃ 83次/分 18次/分 193/76mmHg 176.2cm 70kg 22.5
  - HEENT :
    - Eyes: no anemic conjunctiva, no icteric sclera, normal EOM
    - ENT: no otorrhea, no rhinorrhea, no erythematous mucosa, no injected tonsil, no deformity
  - Neck: supple, freely movable, trachea midline,no deformity, no engorged jugular vein , no palpable thyroid or lymph node enlargement
  - Chest:
    - Inspection: operation scar: skin graft
    - Palpation: no palpable mass or nodule, no subcutaneous emphysema, no tenderness
    - Ausculation: clear breathing sound
  - Heart:
    - Percussion: normal heart size
    - Ausculation: regular heart beat, normal S1, S2, no gallop, no murmur
  - Abdomen:
    - Inspection: in

-
    - flat contour, symetrically, no scar, no stria, no dilated veins, no rashes
  - Ausculation: normoactive bowel sound, no abdominal bruits
  - palpation: soft, no muscle guarding or rigidity, no local tenderness, no rebounding pain, no palpable mass, no palpable pulsation
  - Percussion: no shifting dulllness, no hepatomegaly, no splenomagaly
-

Peripheral pulsation :	CA	BA	RA	FA	PA	PTA	DPA
Right side :	++	++	++	++	++	++	++/+++
Left side :	++	++	++	++	++	++	++/+++

- Genito-anus: no tenderness or palpable mass lesion or rectal shelf, normal external genitalia, normal anal tone, brown stool

CRS CONDITION

- Rectodigital examination: -
- Colonoscope report: UP TO 15 CM FAV. RECTUM TUMOR WAS NOTED AND S/P TATTO.
- CEA: 1.72 ng/ml
-
  - Lung

手術日期及方法

- 手術日期及方法20210107 1. Laparoscopic anterior resection
  2. Intraoperative colonoscopy : (-)
  3. High ligation (+) : At IMA root. Left colic artery and IMV were also ligated
  4. Take down from the splenic flexure : (-)
  5. EndoGIA (60-4.1) X1 + EEA (33 mm) were used
  6. Proximal cut end: X cm, distal cut end: X cm
  7. ADHESION BARRIER FILM was placed below the central abdominal wound.

住院治療經過

- After admission, operation with laparoscopic anterior resection was done on 1/7 as schedule. Under ERAS protocol, we let him try water and then diet as his tolerance. Under the stable condition, he was discharged on 1/10.

合併症

- nil

一般檢查

- 累積報告 :

DATE	TIME	ALB	TP	BIL.T	BIL.D	ALKP	AST	ALT	LDH	CHOL	TG	HDL-C	LDL	UA	NH3	AMY
1100107	1242	3.7		0.46	0.15	75	18	7								

DATE	TIME	WBC	RBC	HGB	HCT	MCV	PLT	BLST	NEUT	BAND	SEG	LYM	MON	EOS	BAS
1100107	1243	7930		9.0	26.9		178		93.3			4.7	1.8	0.1	0.1

DATE	TIME	NA	K	CL	CA	BUN	CREAT	Mg	P	CRP	ESR	PCT	LIPASE	LACTATE
1100107	1242	139	3.8		8.3	15	0.99							

特殊檢查

病理報告

其它

出院時情況

- 3.改門診治療

出院指示

-

藥名	劑量	劑量單位	用法	頻次	數量	數量單位	現況	天數	單位
Ultracet tab	1	TAB	PO	BID&HS	27	TAB	TH	9	天
Mag. Oxide tab	2	TAB	PO	TID	54	TAB	TH	9	天

出院計劃

主治醫師 2062F 蔣鋒帆 / 住院醫師 2331F 陳凱祥

正式摘要(電子簽章：**000000207535041)**

	W52 / W52

doctorRank:B
